# Accuracy of Computer-Guided Dental Implant Placement: A Clinical Comparison of Three Surgical Guide Types

**DOI:** 10.3390/jcm14248652

**Published:** 2025-12-06

**Authors:** Marek Markiewicz, Adam Aleksander Nowicki

**Affiliations:** 1 Markiewicz Clinic, Szymanowskiego 2, 80-280 Gdańsk, Poland; 2 Diamante Clinica Dental Clinic, Sportowa 48 A/C, 59-300 Lubin, Poland; adamusnowicki@gmail.com

**Keywords:** computer-aided implantology, dental implant precision, surgical guide accuracy, guided surgery, static navigation

## Abstract

**Background/Objectives**: The accurate and prosthetically driven placement of dental implants is crucial for long-term clinical success. While computer-aided static navigation enhances precision, the comparative accuracy of different surgical guide support types (teeth-, mucosa-, and bone-supported) under identical clinical conditions remains a critical and less explored variable. This study aimed to compare the accuracy of these three guide types. **Methods**: This clinical study evaluated the precision of computer-aided implant placement by comparing planned versus actual implant positions in fifty participants who received 140 implants. Discrepancies were measured in 3D using STL files in Exocad^®^ DentalCAD software (exocad Elefsina 3.2, Darmstadt, Germany). **Results**: In the maxilla, mean total deviation was 0.443 mm at the implant neck and 0.562 mm at the apex. In the mandible, deviations were higher: 0.755 mm at the neck and 0.981 mm at the apex. Teeth-supported guides demonstrated the highest accuracy. Mucosa-supported guides showed the least precision, particularly in the mandible, while bone-supported guides provided clinically acceptable results. **Conclusions**: Computer-aided static navigation is highly accurate for implant placement. Guide selection should be tailored to anatomical conditions, with bone-supported guides preferred for edentulous mandibles.

## 1. Introduction

The accurate and prosthetically driven placement of dental implants is a cornerstone of their long-term clinical success, influencing functional, aesthetic, and biological outcomes [1,2]. Conventional freehand implant placement, while successful in many cases, is inherently susceptible to deviations from the preoperative plan, potentially leading to complications such as damage to vital anatomical structures or suboptimal prosthetic rehabilitation [3,4].

The advent of computer-aided implantology (CAI) has introduced a paradigm shift, offering enhanced precision through static surgical guides. These guides, fabricated based on a virtual plan that integrates three-dimensional radiographic data (Cone-Beam Computed Tomography, CBCT) and digital surface scans (Standard Tessellation Language, STL), transfer the planned implant position directly to the surgical site [5,6]. Surgical guides are categorized by their support mechanism—teeth-, mucosa-, or bone-supported—each with distinct indications and inherent stability characteristics [7]. The digital workflow and the array of software tools and guided surgery techniques available are extensively reviewed in the literature [8,9]. 

While the overall superiority of guided surgery over freehand methods is well-documented in the literature [10,11,12], a critical and less explored variable is the comparative accuracy of these different guide support types under identical clinical conditions and within a standardized digital workflow. Existing evidence suggests that teeth-supported guides generally offer the highest precision due to rigid fixation [13,14], whereas mucosa-supported guides, particularly in edentulous mandibles, may be prone to larger deviations due to tissue mobility and a lack of stable reference points [15,16,17]. Bone-supported guides, requiring flap reflection, can provide superior stability in atrophic jaws where other reference points are absent [18,19]. However, direct comparative clinical studies evaluating all three guide types across diverse patient scenarios (single-tooth gaps, multiple-tooth gaps, and edentulous jaws) within a single, controlled protocol are limited [20].

Therefore, this clinical study aimed to perform a comparative analysis of the accuracy of implant placement using teeth-, mucosa-, and bone-supported static surgical guides. The primary objective was to quantify and compare the three-dimensional deviations between the planned and achieved implant positions for each guide type, with a specific focus on the challenges posed by edentulous mandibles. The findings are intended to provide clinicians with evidence-based guidance for selecting the most appropriate surgical guide support to optimize clinical outcomes.

## 2. Materials and Methods

### 2.1. Study Design and Ethical Approval

This study was a retrospective, multicenter, observational analysis of data collected between November 2023 and July 2024 from two private dental practices. The analysis was based on standard computer-guided implant treatments, where the use of surgical guides and the type of guide support were determined by clinical necessity as part of routine patient care, without any protocol deviations or additional interventions for the purpose of the study. Consequently, the local Bioethics Committee of the Pomeranian Medical University in Szczecin confirmed that the study did not constitute a medical experiment and therefore did not require its formal approval (KB.006.81.2024, 11 June 2024). This study was reported following the STROBE guidelines for observational studies.

### 2.2. Participants

Fifty systemically healthy patients (26 males and 24 females; mean age 52.54 years) requiring dental implant treatment were included. Inclusion criteria comprised adequate bone volume for implant placement without the need for bone augmentation. Exclusion criteria encompassed uncontrolled systemic diseases (e.g., poorly controlled diabetes, cardiac illness), history of radiotherapy or chemotherapy, bisphosphonate therapy, pregnancy, smoking habit, maxillary parafunctions, and age above 65 years. Participants were consecutively enrolled from the patient population presenting for implant treatment at the two clinics, provided they met the inclusion and exclusion criteria.

Patients were categorized into three clinical groups based on their edentulous status:

Group 1: Single-tooth gaps (up to 4 adjacent gaps; *n* = 20 patients, mean age 44 years).
Group 2: Multiple-tooth gaps (more than 4 adjacent gaps; *n* = 15 patients, mean age 57 years).
Group 3: Fully edentulous arches (*n* = 15 patients, mean age 56 years).

The sample size was determined by the patient pool available for recruitment during the study period at the two participating centers, representing a convenience sample. A post-hoc power analysis was not performed as this was an exploratory observational study aimed at generating hypotheses and describing the precision of different guide types in a real-world clinical setting.

### 2.3. Digital Workflow and Surgical Guide Fabrication

A standardized digital protocol (One Step Concept^®^, Markiewicz Clinic s-ka z o.o., Gdańsk, Poland) was employed for all patients. The workflow commenced with a preliminary CBCT scan (CS 8100, Carestream Dental, Stuttgart, Germany) and intraoral scans (Medit I-500 or I-700, Medit, Seoul, Republic of Korea). For edentulous patients, additional facial scans were acquired to aid in planning.

**Figure 1:**
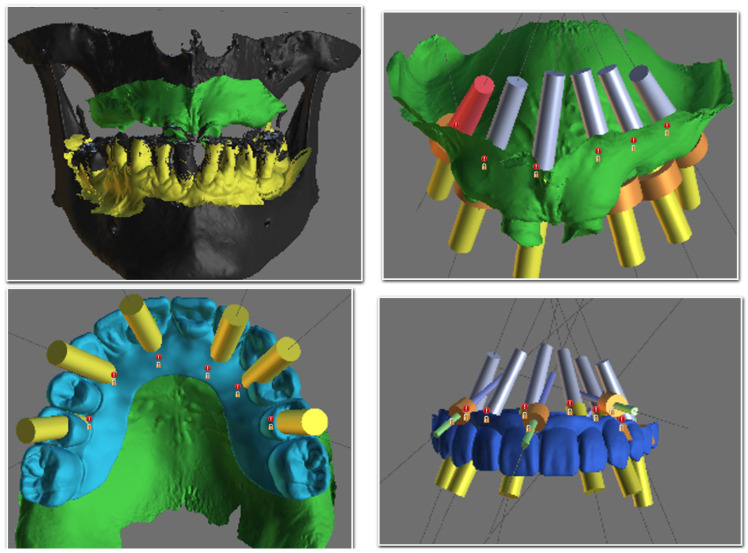
Screenshot from BluSkyBio software (BluSkyPlan module) showing the stages of virtual implant planning.

**Figure 2:**
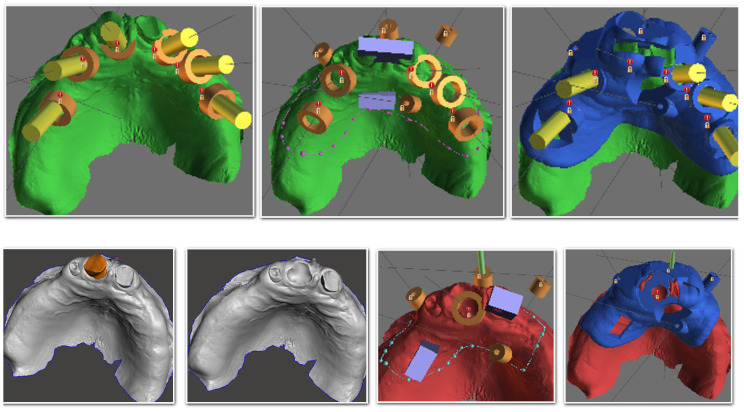
Digital design for surgical guides in BluSkyBio software.

Digital wax-ups were designed, and STL files were imported into planning software (BluSkyBio V4.12, Chicago, IL, USA) for fusion with DICOM data and virtual implant placement. For edentulous cases, a specific protocol involving a relined try-in prosthesis with radiographic markers was used to ensure precise data superimposition. Based on the virtual plan, surgical guides were digitally designed for tooth, mucosa, or bone support (Figure 1 and Figure 2). The guides were fabricated using stereolithography (SLA) with a Phrozen 4 K printer (Phrozen Technology, Hsinchu City, Taiwan) and Nexdent SG resin (Dentsply Sirona, Charlotte, NC, USA), with stainless-steel sleeves incorporated for drill guidance.

### 2.4. Surgical Procedure and Implant Placement

**Figure 3:**
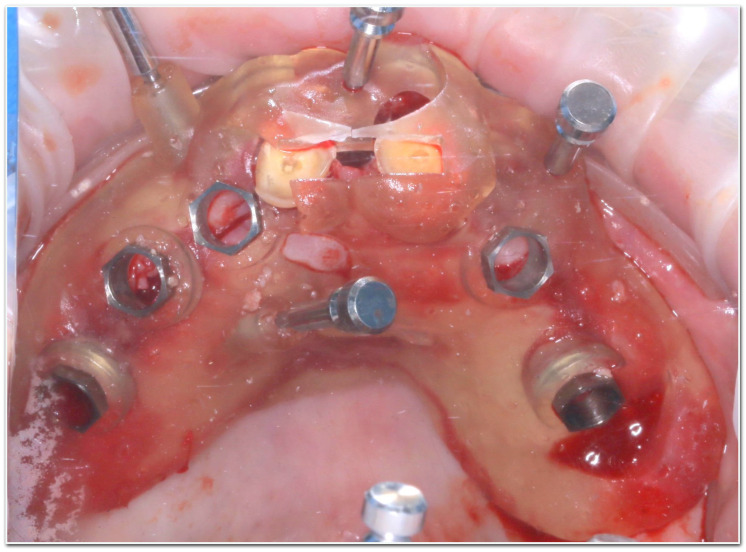
Clinical application of a teeth-supported surgical guide secured on the residual dentition during the osteotomy procedure.

**Figure 4:**
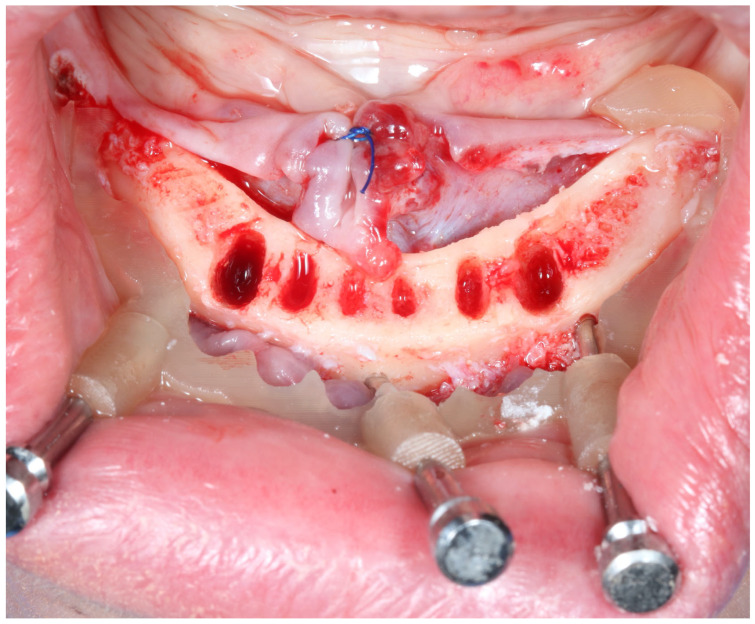
Clinical application of basal part of the bone supported guide.

**Figure 5:**
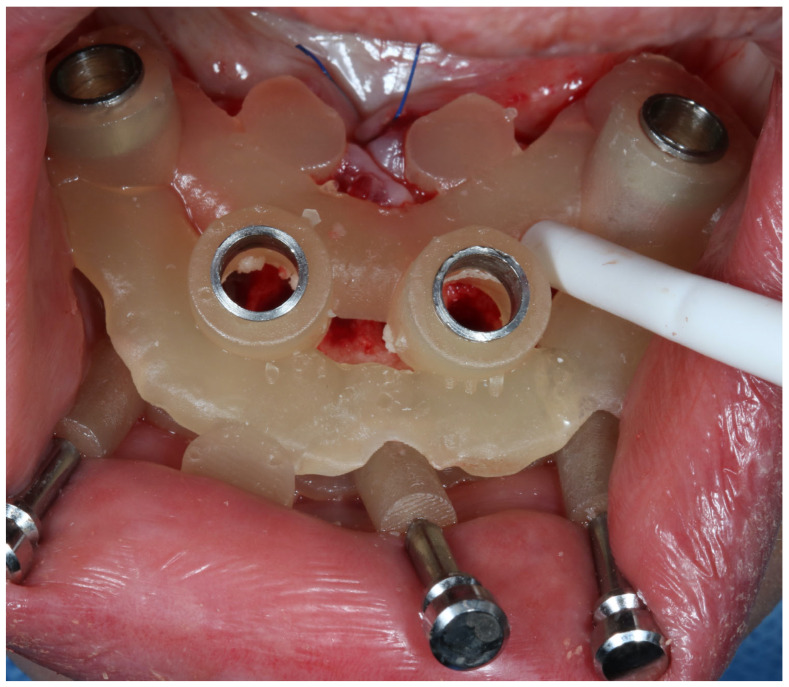
Clinical application of osteotomy part of the bone supported guide.

All surgeries were performed by surgeons of equivalent skill levels using a computer navigation drill kit (Magellan, CMC Equipment, Milan, Italy). Following local anesthesia, the surgical guide was positioned and secured with 3–4 bone fixation pins. A flapless approach was adopted whenever possible; otherwise, a minimal mucoperiosteal flap was elevated. Osteotomies were prepared using the guided drills, and Rootform implants (Pro-Trate, Warszawa, Poland) with diameters ranging from 3.8 mm to 5.5 mm were placed (Figure 3, Figure 4 and Figure 5). A total of 140 implants were inserted (Table 1). Implants were placed with a minimum insertion torque of 35 Ncm and were immediately loaded with a provisional prosthesis.

### 2.5. Outcome Measures and Data Analysis

**Figure 6:**
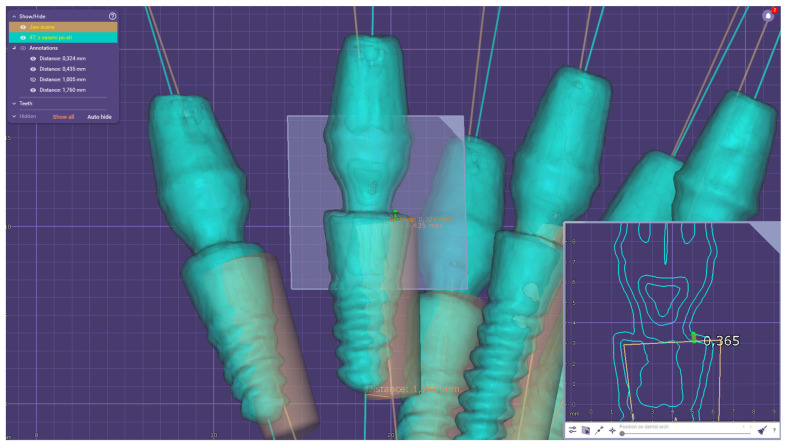
Error measurements in Exocad Software.

**Figure 7:**
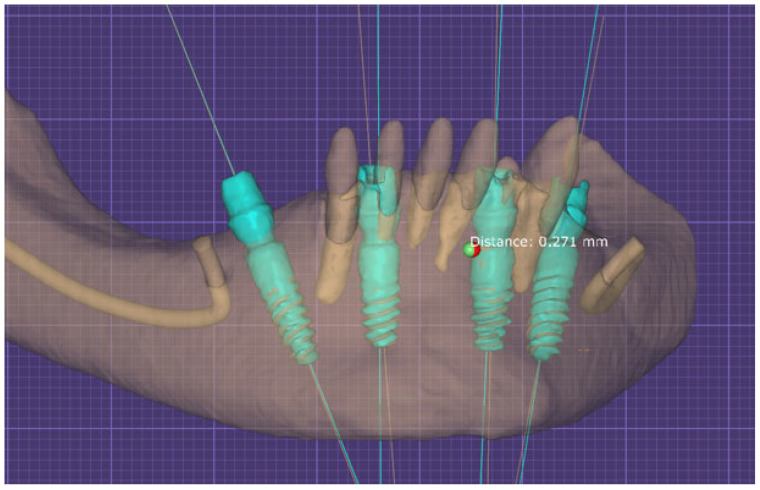
Alignment process of both STL files in Exocad Software.

The primary outcome was the accuracy of implant placement, defined as the three-dimensional deviation between the planned and postoperative implant position. Following implant placement, postoperative CBCT scans were obtained. The DICOM data were segmented, and the resulting STL files of the actual implant positions were aligned with the planning STL files in Exocad^®^ DentalCAD software (exocad Elefsina 3.2, Darmstadt, Germany) (Figure 6 and Figure 7). Measurements were performed for the total deviation at the implant neck (coronal) and apex (apical), as well as the angular deviation (Figure 6). The measurements were conducted twice by two unbiased researchers to assess the consistency and replicability of the data. Findings were categorized based on group type, location within the arch, and patient identity.

To ensure measurement reproducibility, the inter-examiner reliability for the linear and angular measurements between the two researchers was assessed using the Intra-class Correlation Coefficient (ICC). An ICC value of 0.98 was obtained, indicating excellent reliability.

Statistical analysis was performed using Statistica 12 software (TIBCO Software Inc., Palo Alto, CA, USA). The normality of data distribution was assessed with the Shapiro-Wilk test. For comparisons between two groups, the Student’s *t*-test or Mann-Whitney U test was used. For comparisons between three or more groups, one-way ANOVA or the Kruskal-Wallis test was applied, followed by post-hoc tests where appropriate. A significance level of *p* < 0.05 was adopted.

## 3. Results

A total of 140 dental implants were immediately placed in 50 patients using computer-guided static surgery. The distribution of these implants across the maxilla and mandible, stratified by the three clinical groups (single-tooth gaps, multiple-tooth gaps, edentulous), is detailed in Table 1. The posterior maxilla was the most frequent implant site across all groups. No intraoperative complications or implant failures were recorded during the one-year follow-up.

**Scheme 1:**
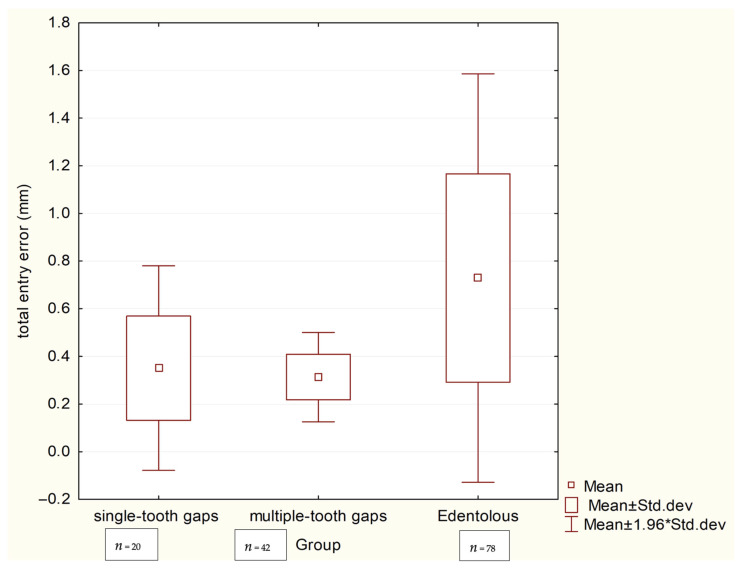
Box plots comparing total coronal deviations (mm) between three groups.

**Scheme 2:**
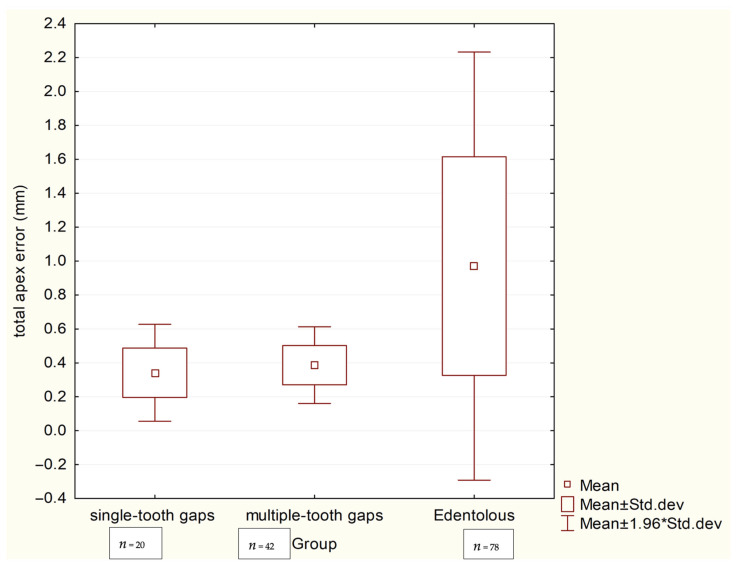
Box plots comparing total apical deviations (mm) between three groups.

The accuracy analysis revealed that the clinical scenario (level of edentulism) significantly influenced precision. As summarized in Table 2, the edentulous group exhibited the largest deviations, with a mean total apex error of 0.971 mm and a coronal error of 0.729 mm. These values were significantly higher (*p* < 0.001) than those in both the single-tooth gaps group (apex: 0.342 mm, coronal: 0.351 mm) and the multiple-tooth gaps group (apex: 0.387 mm, coronal: 0.313 mm), as visually confirmed by the box plots in Scheme 1 and Scheme 2. No significant difference in accuracy was found between the single- and multiple-tooth-gap groups.

Further analysis within each clinical group, considering the implant’s location (anterior/posterior) and jaw (maxilla/mandible), showed no statistically significant regional differences for the single-tooth gaps (Table 3), multiple-tooth gaps (Table 4), or edentulous groups (Table 5).

**Scheme 3:**
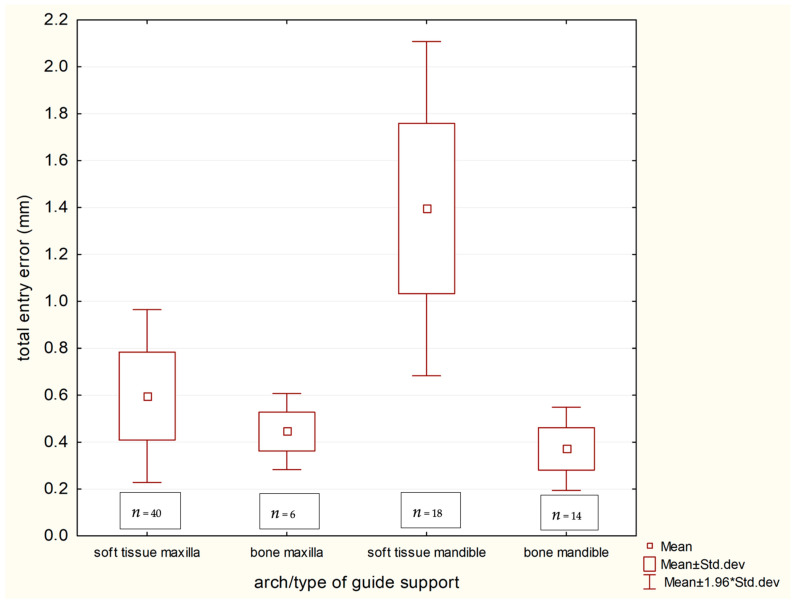
Coronal implant discrepancies in terms of guide support in group nr 3—Edentulous.

**Scheme 4:**
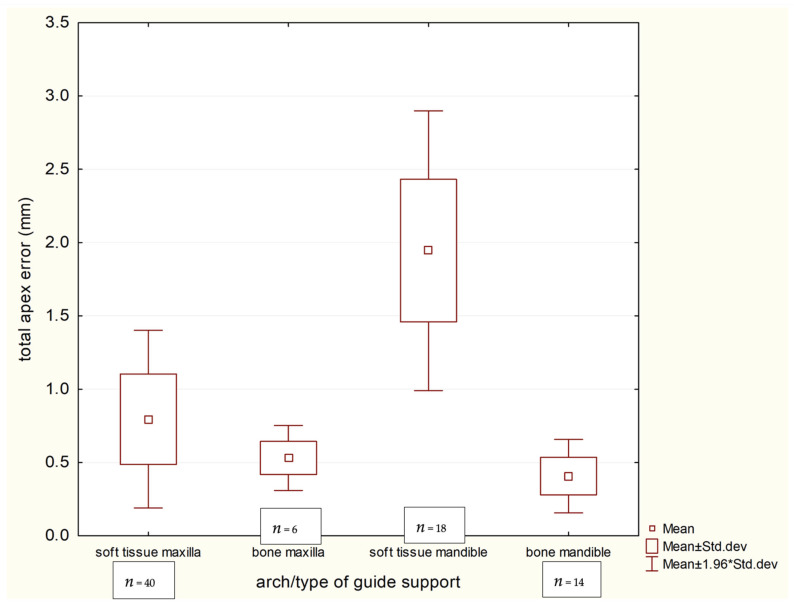
Apical implant discrepancies in terms of guide support in group nr 3—Edentulous.

A pivotal finding of this study pertains to the performance of different guide supports in fully edentulous cases, detailed in Table 6. Mucosa-supported guides in the edentulous mandible resulted in the highest deviations observed in the entire study (coronal: 1.395 mm, apical: 1.945 mm). In stark contrast, bone-supported guides in the edentulous mandible provided markedly superior and clinically excellent accuracy (coronal: 0.371 mm, apical: 0.408 mm), with all pairwise comparisons between these support types in the mandible and maxilla being statistically significant (*p* < 0.01). The profound impact of guide support type in the edentulous arch is clearly demonstrated in Scheme 3 and Scheme 4.

**Scheme 5:**
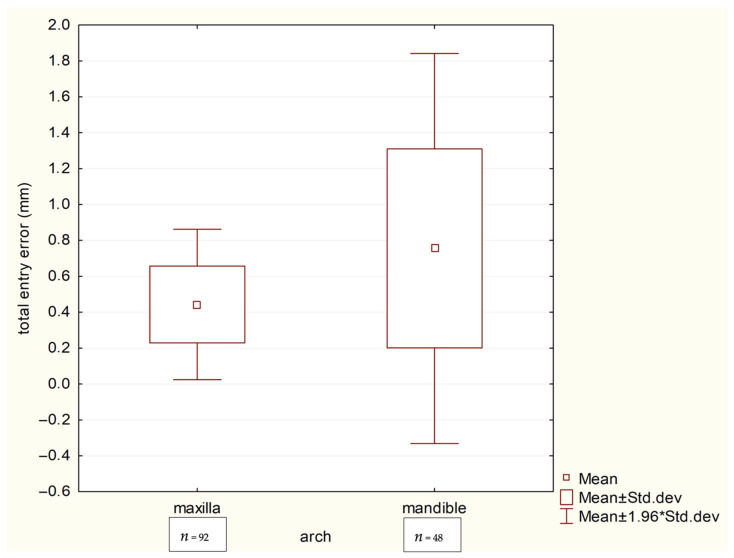
Box plots comparing total coronal deviations (mm) between the maxillary and mandibular arches.

Finally, a direct comparison between the jaws (Table 7 and Table 8, Scheme 5) established that implant placement was significantly more accurate in the maxilla than in the mandible at the coronal level (0.443 mm vs. 0.755 mm, *p* = 0.010). A similar, though statistically non-significant (*p* = 0.086), trend was observed for apical deviations (maxilla: 0.562 mm vs. mandible: 0.981 mm).

## 4. Discussion

Precise preoperative planning is crucial for the successful placement of dental implants, as it must align with restorative objectives and anatomical constraints. Any significant deviations from the planned implant position can adversely affect the prosthetic outcome, resulting in impaired functionality and suboptimal aesthetics. Improper placement of mandibular endosseous implants can potentially damage the inferior alveolar nerve, particularly in edentulous atrophic mandibles [21].

The advantage of navigation technology lies in its ability to accurately control the depth, position, and angle of implant placement. Navigation systems enable meticulous preplanning and precise localization of implant positions, which is especially beneficial for high-risk patients, such as those on anticoagulation therapy or with atrophic mandibles [22]. These systems may also reduce operative time, though the cost-effectiveness of image-based navigation in dental implantology remains to be thoroughly evaluated.

The precision of implant placement is directly influenced by the design and manufacturing of the surgical template. Any inaccuracies in the fabrication process can lead to intraoperative complications. Recent literature extensively evaluates and compares the accuracy of surgical computer-aided navigation techniques. A study by Vercruyssen et al. [23] reported high accuracy, with an error of 0.9 mm (range: 0.1–4.5, mean 0.8) at the neck and 1.2 mm (range: 0.2–4.9, mean 1.1) at the apex, and an angular deviation error of 2.7° (range: 0.0–6.6, mean 2.3). In 2018, Chang-Kai et al. [24] reported horizontal deviation values of 1.50 ± 0.79 mm at the apical endpoint. Additionally, computer-aided static navigation systems showed better placement accuracy (6.02 ± 3.71) than manual placement (9.26 ± 3.62). Hoffmann et al. [25] reported significant differences in implant placement accuracy between computer-aided dynamic navigation systems and manual placement, with mean angular deviations of 4.2 ± 1.8° and 11.2 ± 5°, respectively. Despite methodological and selection criteria differences from the present study, computer-aided dynamic navigation systems demonstrated superior accuracy compared to manual methods. Schneider’s study evaluated implant apex deviation, finding a 1.07 mm deviation at the neck point, 1.63 mm at the apex, and a 5.26° angular discrepancy [26].

Research conducted by Vinci and colleagues identified a discrepancy of 0.30 mm at the apex and a global horizontal discrepancy of 0.37 mm at the neck in the maxilla, while in the mandible, the discrepancy at the apex was 0.43 mm and the global horizontal discrepancy at the neck was 0.28 mm [27]. Such minor angulation and distance differences may be clinically irrelevant in the maxilla.

Our multicenter study, conducted across two private dental practices, confirms that computer-guided implant placement achieves clinically acceptable accuracy, with deviations between virtual planning and actual positioning varying significantly based on anatomical and technical factors. The results demonstrate superior precision in the maxilla, with total apical and coronal deviations of 0.562 mm (95% CI: 0.501–0.623) and 0.443 mm (95% CI: 0.399–0.487), respectively, compared to the mandible’s 0.981 mm (95% CI: 0.758–1.204) at the apex and 0.755 mm (95% CI: 0.592–0.918) at the coronal aspect (Table 7 and Table 8). These arch-specific differences, while statistically significant for coronal deviations (*p* = 0.010), likely reflect the mandible’s anatomical challenges, including dense bone morphology and limited surgical access observed across both clinical sites.

The type of surgical guide support proved to be a critical factor influencing outcomes in both participating practices. Mucosa-supported guides in edentulous cases exhibited the largest deviations (1.395 ± 0.364 mm coronal; 1.945 ± 0.487 mm apical), while bone-supported guides in the edentulous mandible showed markedly better accuracy (0.371 ± 0.090 mm coronal; 0.408 ± 0.128 mm apical) (Table 6). This consistent finding across both clinical sites, validated by robust statistical significance (*p* < 0.001, η^2^ > 0.3 for key comparisons), underscores the universal importance of stable reference points. Teeth-supported guides maintained their reliability in partially edentulous patients at both locations, with deviations as low as 0.313–0.351 mm (Kruskal-Wallis H = 62.47 for coronal, H = 66.00 for apical; both *p* < 0.001). While the reported deviations were statistically significant, their clinical relevance must be considered. The sub-millimeter accuracy achieved with teeth-supported and bone-supported guides is widely regarded as clinically acceptable, falling within the tolerance of modern prosthetic connection systems. However, the deviations exceeding 1.9 mm at the apex with mucosa-supported guides in the mandible approach a magnitude where critical anatomical structures could be compromised, and prosthetic complications become more likely, underscoring a clear clinical significance.

The multicenter design strengthens the clinical relevance of our findings, as the results were consistent across different practice settings and patient populations. This suggests that the observed precision differences based on guide type and arch location are likely to be reproducible in similar clinical environments. For practitioners, these results strongly advocate for protocol adjustments: bone-supported guides should be prioritized in edentulous mandibles, while mucosa-supported templates may be adequate for maxillary cases where anatomical landmarks enhance stability, regardless of the clinical setting.

While our study benefits from the multicenter design, limitations must be acknowledged. The lack of randomization and blinding, inherent to the observational design, could introduce selection bias. The consistent use of a single digital workflow and implant system, while ensuring internal consistency, may affect the generalizability of the absolute deviation values to other protocols. The sample size, though substantial, may limit the power for some subgroup analyses. Furthermore, the one-year follow-up assesses surgical accuracy and immediate loading success but does not evaluate long-term prosthetic outcomes or peri-implant health. To ensure measurement reproducibility, inter-examiner reliability was assessed, yielding an excellent Intra-class Correlation Coefficient (ICC) of 0.98. Nevertheless, the consistency of results across our two practice sites strongly validates static navigation as a reliable tool, provided clinicians carefully select guide types according to each patient’s anatomical context.

Future research should focus on enhancing positioning accuracy and addressing clinical complications, particularly in the challenging context of edentulous mandibles. Emerging technologies, such as augmented reality devices [28,29] and artificial intelligence-assisted planning algorithms [30], hold promise for overcoming current limitations, such as the loss of alveolar ridge visibility during computer-aided navigation procedures, potentially further improving the precision and safety of guided implantology.

## 5. Conclusions

This investigation confirms that computer-aided static navigation is a highly accurate method for dental implant placement, provided the surgical guide is carefully selected based on the patient’s anatomical context. The findings demonstrate that teeth-supported guides provide superior precision in partially edentulous cases (single and multiple tooth gaps). In edentulous cases, the choice of guide support is critical: while mucosa-supported guides perform acceptably in the maxilla, the mandible presents a significant challenge. This study clearly identified that mucosa-supported templates in the mandible yield the poorest outcomes, whereas bone-supported variants provide clinically acceptable accuracy and should be the preferred choice in this demanding clinical scenario. These protocol adjustments, validated across multiple practice environments, are essential to minimize deviations and improve long-term prosthetic outcomes.

## Figures and Tables

**Table 1:** Site positions of implant.

Maxilla			Mandible		
**Total:**	Front Maxilla	33	Total:	Front mandible	17
**Total:**	Back Maxilla	59	Total:	Back Mandible	31
**single-tooth gaps**	Front Maxilla	5	single-tooth gaps	Front mandible	1
	Back Maxilla	8		Back Mandible	6
**multiple-tooth gaps**	Front Maxilla	10	multiple-tooth gaps	Front mandible	5
	Back Maxilla	23		Back Mandible	4
**Edentulous**	Front Maxilla	18	Edentulous	Front mandible	12
	Back Maxilla	28		Back Mandible	20

**Table 2:** Mean implant discrepancies in 3 groups.

	Group	n	Mean	95% CI	Mediana	Min.	Max.	SD
*total entry error* (mm)	single-tooth gaps	20	0.351	0.255–0.447	0.273	0.163	0.928	0.219
*total entry error* (mm)	multiple-tooth gaps	42	0.313	0.284–0.342	0.290	0.150	0.533	0.096
*total entry error* (mm)	Edentulous	78	0.729	0.632–0.826	0.561	0.251	2.167	0.437
*total apex error* (mm)	single-tooth gaps	20	0.342	0.278–0.406	0.299	0.191	0.750	0.146
*total apex error* (mm)	multiple-tooth gaps	42	0.387	0.352–0.422	0.368	0.228	0.818	0.115
*total apex error* (mm)	Edentulous	78	0.971	0.826–1.114	0.671	0.239	2.894	0.644

**Table 3:** Implant discrepancies in group nr 1—Single-tooth gaps.

	Region	n	Mean	Mediana	Min.	Max.	SD
*total entry error* (mm)	Front maxilla	5	0.215	0.216	0.177	0.264	0.039
*total entry error* (mm)	Back mandible	7	0.387	0.325	0.197	0.753	0.196
*total entry error* (mm)	Back maxilla	8	0.404	0.304	0.163	0.928	0.281
*total apex error* (mm)	Front maxilla	5	0.241	0.241	0.191	0.311	0.052
*total apex error* (mm)	Back mandible	7	0.355	0.347	0.248	0.539	0.100
*total apex error* (mm)	Back maxilla	8	0.393	0.324	0.206	0.750	0.192

**Table 4:** Implant discrepancies in group nr 2—Multiple-tooth gaps.

	Region	n	Mean	Mediana	Min.	Max.	SD
*total entry error* (mm)	Back maxilla	23	0.289	0.279	0.150	0.497	0.093
*total entry error* (mm)	Front maxilla	10	0.327	0.281	0.220	0.533	0.107
*total entry error* (mm)	Back mandible	4	0.369	0.371	0.322	0.412	0.041
*total entry error* (mm)	Front mandible	5	0.352	0.362	0.214	0.458	0.104
*total apex error* (mm)	Back maxilla	23	0.369	0.319	0.228	0.818	0.131
*total apex error* (mm)	Front maxilla	10	0.386	0.352	0.268	0.630	0.108
*total apex error* (mm)	Back mandible	4	0.442	0.447	0.373	0.502	0.064
*total apex error* (mm)	Front mandible	5	0.423	0.405	0.345	0.554	0.082

**Table 5:** Implant discrepancies in group nr 3—Edentulous.

	Region	n	Mean	Mediana	Min.	Max.	SD
*total entry error* (mm)	Back maxilla	28	0.572	0.533	0.346	1.085	0.182
*total entry error* (mm)	Front maxilla	18	0.584	0.544	0.366	1.151	0.193
*total entry error* (mm)	Back mandible	20	0.953	1.022	0.251	2.167	0.577
*total entry error* (mm)	Front mandible	12	0.939	0.762	0.349	2.008	0.625
*total apex error* (mm)	Back maxilla	28	0.779	0.649	0.412	1.751	0.320
*total apex error* (mm)	Front maxilla	18	0.732	0.641	0.455	1.447	0.283
*total apex error* (mm)	Back mandible	20	1.279	1.483	0.239	2.894	0.839
*total apex error* (mm)	Front mandible	12	1.261	1.095	0.269	2.815	0.927

**Table 6:** Implant discrepancies in terms of guide support in group nr 3—Edentulous.

	Arch/Type of Guide Support	n	Mean	95%Cl	Mediana	Min.	Max.	SD
*total entry error (mm)*	soft tissue maxilla	40	0.596	0.538–0.654	0.561	0.346	1.151	0.188
*total entry error (mm)*	bone maxilla	6	0.445	0.379–0.511	0.438	0.349	0.556	0.083
*total entry error (mm)*	soft tissue mandible	18	1.395	1.227–1.563	1.315	0.912	2.167	0.364
*total entry error (mm)*	bone mandible	14	0.371	0.324–0.418	0.360	0.251	0.599	0.090
*total apex error (mm)*	soft tissue maxilla	40	0.795	0.699–0.891	0.662	0.504	1.751	0.309
*total apex error (mm)*	bone maxilla	6	0.531	0.441–0.621	0.496	0.412	0.704	0.113
*total apex error (mm)*	soft tissue mandible	18	1.945	1.720–2.170	1.931	1.264	2.894	0.487
*total apex error (mm)*	bone mandible	14	0.408	0.341–0.475	0.428	0.239	0.715	0.128

**Table 7:** Total entry implant discrepancies.

	Arch	n	Mean	95%Cl	Mediana	Min.	Max.	SD
*total entry error* (mm)	maxilla	92	0.443	0.399–0.487	0.425	0.150	1.151	0.214
*total entry error* (mm)	mandible	48	0.755	0.598–0.912	0.442	0.197	2.167	0.554

**Table 8:** Total apex implant discrepancies.

	Arch	n	Mean	95%Cl	Mediana	Min.	Max.	SD
*total apex error* (mm)	maxilla	92	0.562	0.499–0.625	0.526	0.191	1.751	0.308
*total apex error* (mm)	mandible	48	0.981	0.751–1.211	0.487	0.239	2.894	0.814

## Data Availability

The data presented in this study are available on request from the corresponding author. The data are not publicly available due to privacy and ethical restrictions.

## Abbreviations

CBCT	Cone Beam Computed Tomography
STL	Standard Tessellation Language (3D file format for CAD/CAM)
DICOM	Digital Imaging and Communications in Medicine (medical imaging standard)
CAD	Computer-Aided Design
CAM	Computer-Aided Manufacturing
SLA	Stereolithography (3D printing technology)
ANOVA	Analysis of Variance (statistical method)
ICC	Intra-class Correlation Coefficient
